# Supplementary material for: Generative AI as a Tool for Environmental Health Research Translation
Source: medRxiv. 2023 Feb 22:2023.02.14.23285938. Preprint. [Version 1] doi: 10.1101/2023.02.14.23285938 (PMC9980240; doi:10.1101/2023.02.14.23285938)
Supplement: Supplement 1 [file NIHPP2023.02.14.23285938v1-supplement-1.pdf]

medRxiv preprint doi: https://doi.org/10.1101/2023.02.14.23285938; this version posted February 22, 2023. The copyright holder for this preprint (which was not certified by peer review) is the author/funder, who has granted medRxiv a license to display the preprint in perpetuity.
It is made available under a CC-BY 4.0 International license .

Supplemental Material

Generative AI as a Tool for Environmental Health Research Translation

Lauren B. Anderson,[1,2] Dhiraj Kanneganti,[1] Mary Bentley Houk,[1] Rochelle H Holm[1] and Ted Smith[1,3*]

[1]Christina Lee Brown Envirome Institute, School of Medicine, University of Louisville, Louisville, KY 40202, United States
[2]Department of Urban and Public Affairs, College of Arts and Sciences, University of Louisville, Louisville KY 40208
[3]University of Louisville Superfund Research Center, Louisville, KY 40202, United States

Correspondence
Correspondence should be sent to Ted Smith, Superfund Research Center, School of Medicine, University of Louisville, 302 E Muhammad Ali Blvd, Louisville, KY 40202 (e-mail: ted.smith@louisville.edu).

medRxiv preprint doi: https://doi.org/10.1101/2023.02.14.23285938; this version posted February 22, 2023. The copyright holder for this preprint (which was not certified by peer review) is the author/funder, who has granted medRxiv a license to display the preprint in perpetuity.
It is made available under a CC-BY 4.0 International license .

List of peer-reviewed research papers authored by University of Louisville Envirome Institute environmental health investigators and collaborating research partners entered into the ChatGPT interface:

1. McLeish AC, Smith T, Riggs DW, Hart JL, Walker KL, Keith RJ, et al. 2022. Community□based evaluation of the associations between well□being and cardiovascular disease risk. Journal of the American Heart Association 11(22):e027095. https://doi.org/10.1161/JAHA.122.027095
2. El-Mallakh TV, Hedges S, Rai JP, Bhatnagar A, Moyer S, El-Mallakh RS. 2022. Suicide and homicide more common with limited urban tree canopy cover. Cities and the Environment 14(2):4. https://doi.org/10.15365/cate.2022.140204
3. Pfeiffer JA, Hart JL, Wood LA, Bhatnagar A, Keith RJ, Yeager RA, et al. 2021. The importance of urban planning: Views of greenness and open space is reversely associated with self-reported views and depressive symptoms. Population medicine 3:20. https://doi.org/10.18332/popmed/139173
4. Coleman CJ, Yeager RA, Riggs DW, Coleman NC, Garcia GR, Bhatnagar A, et al. 2021. Greenness, air pollution, and mortality risk: A US cohort study of cancer patients and survivors. Environment International 157:106797. https://doi.org/10.1016/j.envint.2021.106797
5. Coleman CJ, Yeager RA, Pond ZA, Riggs DW, Bhatnagar A, Pope III CA. 2022. Mortality risk associated with greenness, air pollution, and physical activity in a representative US cohort. Science of The Total Environment 824:153848. https://doi.org/10.1016/j.scitotenv.2022.153848

Table S1. Evaluation responses from studied peer-reviewed papers

	**Coleman CJ, Yeager RA, Pond ZA, Riggs DW, Bhatnagar A, Pope III CA. 2022. Mortality risk associated with greenness, air pollution, and physical activity in a representative US cohort. Science of The Total Environment 824:153848. https://doi.org/10.1016/j.scitotenv.2022.153848**	**Coleman CJ, Yeager RA, Riggs DW, Coleman NC, Garcia GR, Bhatnagar A, et al. 2021. Greenness, air pollution, and mortality risk: A US cohort study of cancer patients and survivors. Environment International 157:106797. https://doi.org/10.1016/j.envint.2021.106797**	**El-Mallakh TV, Hedges S, Rai JP, Bhatnagar A, Moyer S, El-Mallakh RS. 2022. Suicide and homicide more common with limited urban tree canopy cover. Cities and the Environment 14(2):4. https://doi.org/10.15365/cate.2022.140204**	**McLeish AC, Smith T, Riggs DW, Hart JL, Walker KL, Keith RJ, et al. 2022. Community based evaluation of the associations between well being and cardiovascular disease risk. Journal of the American Heart Association 11(22):e027095. https://doi.org/10.1161/JAHA.122.027095**	**Pfeiffer JA, Hart JL, Wood LA, Bhatnagar A, Keith RJ, Yeager RA, et al. 2021. The importance of urban planning: Views of greenness and open space is reversely associated with self-reported views and depressive symptoms. Population medicine 3:20. https://doi.org/10.18332/popmed/139173**
500 Word summary accuracy	3	1	4	4	3
500 Word summary completeness	2	3	4	5	4
500 Word summary readability	4	3	4	3	4
500 Word summary acceptable for public (1=Y, 0=N)	1	0	1	1	0

medRxiv preprint doi: https://doi.org/10.1101/2023.02.14.23285938; this version posted February 22, 2023. The copyright holder for this preprint (which was not certified by peer review) is the author/funder, who has granted medRxiv a license to display the preprint in perpetuity.
It is made available under a CC-BY 4.0 International license .

	**Coleman CJ, Yeager RA, Pond ZA, Riggs DW, Bhatnagar A, Pope III CA. 2022. Mortality risk associated with greenness, air pollution, and physical activity in a representative US cohort. Science of The Total Environment 824:153848. https://doi.org/10.1016/j.scitotenv.2022.153848**	**Coleman CJ, Yeager RA, Riggs DW, Coleman NC, Garcia GR, Bhatnagar A, et al. 2021. Greenness, air pollution, and mortality risk: A US cohort study of cancer patients and survivors. Environment International 157:106797. https://doi.org/10.1016/j.envint.2021.106797**	**El-Mallakh TV, Hedges S, Rai JP, Bhatnagar A, Moyer S, El-Mallakh RS. 2022. Suicide and homicide more common with limited urban tree canopy cover. Cities and the Environment 14(2):4. https://doi.org/10.15365/cate.2022.140204**	**McLeish AC, Smith T, Riggs DW, Hart JL, Walker KL, Keith RJ, et al. 2022. Community□based evaluation of the associations between well□being and cardiovascular disease risk. Journal of the American Heart Association 11(22):e027095. https://doi.org/10.1161/JAHA.122.027095**	**Pfeiffer JA, Hart JL, Wood LA, Bhatnagar A, Keith RJ, Yeager RA, et al. 2021. The importance of urban planning: Views of greenness and open space is reversely associated with self-reported views and depressive symptoms. Population medicine 3:20. https://doi.org/10.18332/popmed/139173**
500 Word summary comments	This summary is focusing on a lot of the methods without too much focus on the actual findings. And the limitations listed are not actually limitations of the study, but a summary of the null findings.	There are several major misinterpretations of the study. 1)The title of the paper is not correct. 2)The study is actually an individual level analysis, with each individuals exposure levels estimated at the county level. 3)The study did not find a decrease in cardiopulmonary mortality associated with greenness in the full cohort of cancer patients, but they did find an association when stratifying to individuals with high survivability cancers. It also should have been noted that this is a study of cancer survivors, and the results do not necessarily apply to the general population.	It's repetitive	Potential of CVD and CVD risk. The language is still a little to technical/complex	Readability depends on audience (I assigned a 4 assuming an academic audience; if audience is general/community members, I'd shift that rating to a 3).

medRxiv preprint doi: https://doi.org/10.1101/2023.02.14.23285938; this version posted February 22, 2023. The copyright holder for this preprint (which was not certified by peer review) is the author/funder, who has granted medRxiv a license to display the preprint in perpetuity.
It is made available under a CC-BY 4.0 International license .

	**Coleman CJ, Yeager RA, Pond ZA, Riggs DW, Bhatnagar A, Pope III CA. 2022. Mortality risk associated with greenness, air pollution, and physical activity in a representative US cohort. Science of The Total Environment 824:153848. https://doi.org/10.1016/j.scitotenv.2022.153848**	**Coleman CJ, Yeager RA, Riggs DW, Coleman NC, Garcia GR, Bhatnagar A, et al. 2021. Greenness, air pollution, and mortality risk: A US cohort study of cancer patients and survivors. Environment International 157:106797. https://doi.org/10.1016/j.envint.2021.106797**	**El-Mallakh TV, Hedges S, Rai JP, Bhatnagar A, Moyer S, El-Mallakh RS. 2022. Suicide and homicide more common with limited urban tree canopy cover. Cities and the Environment 14(2):4. https://doi.org/10.15365/cate.2022.140204**	**McLeish AC, Smith T, Riggs DW, Hart JL, Walker KL, Keith RJ, et al. 2022. Community□based evaluation of the associations between well□being and cardiovascular disease risk. Journal of the American Heart Association 11(22):e027095. https://doi.org/10.1161/JAHA.122.027095**	**Pfeiffer JA, Hart JL, Wood LA, Bhatnagar A, Keith RJ, Yeager RA, et al. 2021. The importance of urban planning: Views of greenness and open space is reversely associated with self-reported views and depressive symptoms. Population medicine 3:20. https://doi.org/10.18332/popmed/139173**
8th Grade summary accuracy	3	4	5	3	3
8th Grade summary completeness	3	4	5	5	3
8th Grade summary readability	4	4	5	4	4
8th Grade summary acceptable for public (1=Y, 0=N)	1	1	1	1	1
8th grade summary comments	There are some minor inaccuracies. The survey did not ask about the level of pollution or how green the area around their home was.			Was no finding on CVD but findings on CVD risk	
Most important finding accuracy	4	3	4	5	4
Most important finding completeness	4	4	4	5	4
Most important finding readability	5	5	4	3	4

medRxiv preprint doi: https://doi.org/10.1101/2023.02.14.23285938; this version posted February 22, 2023. The copyright holder for this preprint (which was not certified by peer review) is the author/funder, who has granted medRxiv a license to display the preprint in perpetuity.
It is made available under a CC-BY 4.0 International license .

	**Coleman CJ, Yeager RA, Pond ZA, Riggs DW, Bhatnagar A, Pope III CA. 2022. Mortality risk associated with greenness, air pollution, and physical activity in a representative US cohort. Science of The Total Environment 824:153848. https://doi.org/10.1016/j.scitotenv.2022.153848**	**Coleman CJ, Yeager RA, Riggs DW, Coleman NC, Garcia GR, Bhatnagar A, et al. 2021. Greenness, air pollution, and mortality risk: A US cohort study of cancer patients and survivors. Environment International 157:106797. https://doi.org/10.1016/j.envint.2021.106797**	**El-Mallakh TV, Hedges S, Rai JP, Bhatnagar A, Moyer S, El-Mallakh RS. 2022. Suicide and homicide more common with limited urban tree canopy cover. Cities and the Environment 14(2):4. https://doi.org/10.15365/cate.2022.140204**	**McLeish AC, Smith T, Riggs DW, Hart JL, Walker KL, Keith RJ, et al. 2022. Community□based evaluation of the associations between well□being and cardiovascular disease risk. Journal of the American Heart Association 11(22):e027095. https://doi.org/10.1161/JAHA.122.027095**	**Pfeiffer JA, Hart JL, Wood LA, Bhatnagar A, Keith RJ, Yeager RA, et al. 2021. The importance of urban planning: Views of greenness and open space is reversely associated with self-reported views and depressive symptoms. Population medicine 3:20. https://doi.org/10.18332/popmed/139173**
Most important finding acceptable for public (1=Y, 0=N)	1	1	1	1	1
Most important finding comments		The study found that greenness only protects against cardiopulmonary mortality in individuals with high survivable cancers, not for the full study.		A little too technical	Generally usable with public (but less so on last sentence, especially last phrase)
Real world impacts accuracy	4	4	4	2	5
Real world impacts completeness	4	4	4	2	4
Real world impacts readability	5	5	4	4	4
Real world impacts acceptable for public (1=Y, 0=N)	1	1	1	0	1

medRxiv preprint doi: https://doi.org/10.1101/2023.02.14.23285938; this version posted February 22, 2023. The copyright holder for this preprint (which was not certified by peer review) is the author/funder, who has granted medRxiv a license to display the preprint in perpetuity.
It is made available under a CC-BY 4.0 International license .

	**Coleman CJ, Yeager RA, Pond ZA, Riggs DW, Bhatnagar A, Pope III CA. 2022. Mortality risk associated with greenness, air pollution, and physical activity in a representative US cohort. Science of The Total Environment 824:153848. https://doi.org/10.1016/j.scitotenv.2022.153848**	**Coleman CJ, Yeager RA, Riggs DW, Coleman NC, Garcia GR, Bhatnagar A, et al. 2021. Greenness, air pollution, and mortality risk: A US cohort study of cancer patients and survivors. Environment International 157:106797. https://doi.org/10.1016/j.envint.2021.106797**	**El-Mallakh TV, Hedges S, Rai JP, Bhatnagar A, Moyer S, El-Mallakh RS. 2022. Suicide and homicide more common with limited urban tree canopy cover. Cities and the Environment 14(2):4. https://doi.org/10.15365/cate.2022.140204**	**McLeish AC, Smith T, Riggs DW, Hart JL, Walker KL, Keith RJ, et al. 2022. Community‐based evaluation of the associations between well‐being and cardiovascular disease risk. Journal of the American Heart Association 11(22):e027095. https://doi.org/10.1161/JAHA.122.027095**	**Pfeiffer JA, Hart JL, Wood LA, Bhatnagar A, Keith RJ, Yeager RA, et al. 2021. The importance of urban planning: Views of greenness and open space is reversely associated with self-reported views and depressive symptoms. Population medicine 3:20. https://doi.org/10.18332/popmed/139173**
Real word impacts comments			Would edit to mention importance of increasing tree canopy, and not just green spaces	They missed the application that was discussed on the paper	

medRxiv preprint doi: https://doi.org/10.1101/2023.02.14.23285938; this version posted February 22, 2023. The copyright holder for this preprint (which was not certified by peer review) is the author/funder, who has granted medRxiv a license to display the preprint in perpetuity.
It is made available under a CC-BY 4.0 International license .
